# Supplementary material for: Augmented CO2 tolerance by expressing a single H+-pump enables microalgal valorization of industrial flue gas
Source: Nat Commun. 2021 Oct 18;12:6049. doi: 10.1038/s41467-021-26325-5 (PMC8523702; doi:10.1038/s41467-021-26325-5)
Supplement: Supplementary file 1 — Supplementary Information [file 41467_2021_26325_MOESM1_ESM.pdf]

Augmented CO_2 tolerance by expressing a single H^+-pump enables microalgal valorization of industrial flue gas

Choi *et al.*

Supplementary Note 1. Comparison of the specific growth rate and concomitant pH of *C. reinhardtii* culture under high CO_2-related stress conditions: Prediction of factor influencing high CO_2-derived toxicity

The provision of a high concentration of CO_2 provokes drastic alterations in various abiotic factors of liquid algal culture. The dissolution of enriched CO_2 mainly triggers acidification of the extracellular milieu and an increase in the concentration of dissolved inorganic carbon (DIC). Given these extracellular environmental changes, high [H^+] and [DIC] could be considered possible causes of the decrease in cellular viability attributed to high CO_2. Because the former could lead to a reduction in the intracellular pH (pH_i) by the influx of H^+ into the cell and the latter could cause osmotic stress to the cell. However, the identity of the dominant toxic factor that causes cell growth inhibition under high CO_2 conditions has not been experimentally demonstrated. To investigate the effects of putative factors on cell growth, we observed the specific growth rates (μ; d^{-1}) of a CO_2-intolerant microalga, *C. reinhardtii*, exposed to extremely high CO_2 conditions (20% CO_2-enriched air) as a proxy for cell viability. At the same time, those of the cells grown under corresponding pH (pH 6.2) or DIC concentration (7 mM) conditions were independently monitored to evaluate the effects of each possible factor. A [DIC] of 7 mM was selected based on a previous study that elucidated the effect of the gaseous CO_2 concentration on the [DIC] in the liquid phase.[1]

The growth phase could be divided into three different stages according to shifts in the external pH (pH_e) (Supplementary Fig. 4). Unlike Stage 2, Stage 1 and Stage 3 showed relatively sudden changes in the pH_e, and these can be attributed to the rapid dissolution of acidic gas (*i.e.,* CO_2) and ammonium (NH_4^+) uptake by cells, respectively.[1] The nonbuffered control group clearly demonstrated the acidifying effect of NH_4^+ uptake as the cell population continued to increase (Supplementary Fig. 4e). Thus, Stage 2 was chosen as an appropriate period to compare the effect of each factor while excluding the influences of other variables, such as radical pH shifts. Notably, NH_4^+ uptake-related acidification in this experimental system is inevitable since NH_4^+ must be provided as a nitrogen source because *C. reinhardtii* CC-125, the microalgal strain used in this study, carries mutations in *nit1* and *nit2* that deprive the cell of the ability to utilize other nitrogen sources, such as nitrate.[2]

As expected, the algal cells exposed to high CO_2 showed a significantly reduced μ value during Stage 2 compared with the cells grown under ambient conditions (Table 1). In contrast, such serious negative effects on

cell viability were not observed during Stage 2 with the cells exposed to correspondingly low pH and high [DIC] conditions. The low pH condition only caused slight growth retardation, whereas the high [DIC] improved the growth rate. The unexpected growth improvement probably occurred because the supplemented DIC was beneficially utilized as an inorganic carbon source for cell growth.[3] As a balance, the changes in the external environment induced by a high concentration of CO_2 (*e.g.*, low pH and high [DIC]) may not be the only and direct factor for the severe levels of pH_i acidification that are an immediate cause of high CO_2-derived cellular toxicity.

Supplementary Method 1. Colony PCR

To isolate and confirm the insertion of the transgene, colony PCR was performed.[4] Candidate cell lines possessing Zeocin resistance (*i.e.*, colonies appearing on the selective medium) were individually picked and transferred to liquid TAP medium supplemented with Zeocin (5 μg mL^{-1}) in a 96-well plate. After 2 days of incubation (at 23 °C under 50 μE m^{-2} s^{-1} light conditions), the cells were collected through centrifugation at 2,866 xg. The cell pellet was resuspended in 50 μL of 5% (w/v) Chelex-100 (Bio-Rad, USA). The suspension was then vigorously vortexed and heated at 95 °C for 10 min. Subsequently, the resulting lysate was immediately chilled on ice. The sample was vortexed for 1 min, and cell debris was separated by centrifugation at 2,866 xg. The genomic DNA-containing supernatant was directly used as a template. The sequence information of the designed primer sets is described in Supplementary Table 1. During investigation of the insertion of the PMA4ΔCter-V gene, an endogenous control gene (IDA5; an actin protein) of the alga was also amplified.[5] Takara PrimeSTAR® GXL DNA polymerase (Takara, Japan) was employed for PCR. A the three-step process consisting of 35 cycles (denaturation at 98 °C for 15 s; annealing at 62 °C for 15 s; and extension at 68 °C for 5 min) was used and 5 min preactivation and 15 min final extension steps were additionally implemented at the beginning and end of the thermal cycling process, respectively.

Supplementary Method 2. Identification of the transgene insertion site

Genomic DNA was extracted and purified using a DNeasy Plant Mini Kit (Qiagen, Germany). The transgene insertion location was detected based on whole genome resequencing.[4] First, the quantity and quality of extracted genomic DNA were determined spectrophotometrically using a NanoDrop™ device (Thermo Fisher Scientific, USA) and agarose gel-based electrophoresis, respectively. Following the examination, DNA libraries were constructed using a TruSeq Nano DNA Sample Preparation Kit (Illumina, USA) according to the manufacturer's instructions. The libraries were sequenced with 150-base paired-end reads using a NovaSeq™ 6000 sequencing system (Illumina, USA). Using HISAT2 software, all reads were mapped to the inserted sequence (PMA4ΔCter-V gene). Subsequently, the reads that partially matched the target sequence (*i.e.*, soft clipped reads) were collected and the selected reads were realigned to the *C. reinhardtii* reference genome (NCBI), which enabled identification of the insert junction. The gene integration site was then confirmed by PCR with specific primer sets (Supplementary Figs. 8b–8c).

Supplementary Method 3. Quantitative real-time PCR

To confirm the expression of PMA2, PMA3, and PMA4ΔCter-V at the mRNA level, total RNA was extracted from the algal cell lines as described above. The quantity and quality of the isolated RNA were also examined using a NanoDrop™ device (Thermo Fisher Scientific, USA) as mentioned above. The extracted RNA was immediately reverse transcribed into cDNA using the QuantiTect® Reverse Transcription Kit (Qiagen, Germany) following the manufacturer's instructions. qRT-PCR was then conducted with the QuantiFast™ SYBR® Green PCR kit (Qiagen, Germany) using the Rotor-Gene Q 2plex platform (Qiagen, Germany). Information on the primer sets used for quantifying PMA2, PMA3, PMA4ΔCter-V and actin (as a housekeeping gene) gene expression is provided in Supplementary Table 1. A three-step amplification process consisting of 40 cycles (denaturation at 95 °C for 15 s; annealing at 55 °C for 25 s; and extension at 72 °C for 30 s) was employed, and a DNA polymerase preactivation step (95 °C for 15 min) preceded the thermal cycling process. The relative expression level of PMA4ΔCter-V was measured based on the conventional $2^{-\Delta\Delta Ct}$ method.

Supplementary Method 4. Western blot

To verify the condition-dependent PMA expression at the protein level in the WT strain, equal numbers of cells ($\sim 2 \times 10^7$) from each condition were harvested and the resulting cell pellet was resuspended in a mixture of NuPAGE™ LDS Sample Buffer (4×) and NuPAGE™ Reducing Agent (10× DTT; final concentration of 50 mM) at an appropriate dilution as indicated in the manufacturer's protocol (Thermo Fisher Scientific, USA). Subsequently, the mixture was incubated by gentle thermal treatment at 70 °C for 10 min to obtain total protein extracts from each sample. After the extract was treated with Halt™ Protease Inhibitor Cocktail (Thermo Fisher Scientific, USA), the total protein concentration was quantified with a Pierce™ BCA Protein Assay kit (Thermo Fisher Scientific, USA). Extract amounts containing the same amount of total protein (200 µg) were then loaded into a NuPAGE™ Bis-Tris Gel with 4–12% polyacrylamide gradient. The gel was run at 100 V for 150 min at 4 ºC. The separated proteins according to the molecular weights were electrotransferred (20 V for 60 min) to a positively charged polyvinylidene difluoride (PVDF) membrane that was preactivated by methyl alcohol treatment. The membrane was blocked for 1 h in TBS-T (20 mM Tris-HCl adjusted to pH 7.6, 137 mM NaCl, 0.1% v/v Tween-20) with 5% (w/v) skim milk at room temperature. The membrane was then immunotreated overnight with 1:1000 and 1:10,000 diluted primary antibodies against plant H^+ ATPase (AS07 260; Agrisera, Sweden) and beta-subunit of ATP synthase (AtpB) (AS05 085; Agrisera, Sweden) derived from rabbit, respectively, in TBS-T

solution at 4 °C and then subjected to a 1 h incubation with 1:100,000 diluted anti-rabbit horseradish peroxidase-conjugated secondary antibody (AS09 602; Agrisera, Sweden) in TBS-T solution at room temperature. The membrane was washed three times with the TBS-T solution at room temperature between each antibody treatment with rocking at 50 rpm. The immunoblot was developed with SuperSignal™ West Femto Maximum Sensitivity Substrate (Thermo Fisher Scientific, USA) during a 5 min incubation. Images were taken using ImageQuant™ LAS 4000 mini (GE Healthcare, USA) (high resolution mode, 40 s exposure). ImageJ software was used for quantification of the PMA protein expression. The intensity of the major band of the native PMA was normalized to that of the loading control (*i.e.*, AtpB). The abovementioned conditions for the sample preparation and detection (*e.g.*, the amount of sample loaded and the use of the highly sensitive reagents) were chosen considering the extracellular condition-dependent protein expression can possibly cause the low abundance of the native PMA in the total protein samples.

On the one hand, to detect the exogenous protein, equal numbers of cells ($\sim 1.25 \times 10^7$) from each strain (WT, PMA4ΔCter-V4, and PMA4ΔCter-V10) grown autotrophically under ambient CO_2 conditions were harvested and resuspended in 1 mL of buffer solution (HEPES 10 mM, NaCl 100 mM, pH adjusted to 7.0). A total of 800 μL of the cell suspension was blended with 5× sample loading buffer consisting of 250 mM Tris-HCl adjusted to pH 6.8, 10% sodium dodecyl sulfate (SDS; w/v), 0.5% bromophenol blue (w/v), 50% glycerol (v/v), and 5% β-mercaptoethanol. Subsequently, the mixture was incubated by gentle thermal treatment at 60 °C for 2 h, which yielded the crude protein extract. 20 μL of the protein extract (corresponding to 20 μg of total protein) was loaded onto a Tris-glycine gel containing 8% (w/v) acrylamide. The gel was run at 100 V for 90 min at room temperature. The separated proteins according to the molecular weights were electrotransferred (100 V for 60 min) to a positively charged polyvinylidene difluoride membrane that was preactivated by methyl alcohol treatment. The membrane was blocked for 2 h in TBS-T with 5% (w/v) skim milk at room temperature. Then, the membrane was immunotreated overnight with 1:1000 and 1:10,000 diluted primary antibodies against plant H^+ ATPase (AS07 260; Agrisera, Sweden) and beta-subunit of ATP synthase (AtpB) (AS05 085; Agrisera, Sweden) derived from rabbit, respectively, in TBS-T solution at 4 °C, followed by a 1 h incubation with a 1:1500 diluted anti-rabbit horseradish peroxidase-conjugated secondary antibody (AS09 602; Agrisera, Sweden) in TBS-T solution at room temperature. Using the TBS-T solution, the membrane was washed three times at room temperature in the interval of each antibody treatment with rocking at 50 rpm. The immunoblot was developed with the PicoEPD Western

Reagent Kit (ELPIS Biotech, Republic of Korea) for 1 min and exposed to the X-ray film for visualization for 5 min.

Supplementary Method 5. Localization of protein

Prior to all the localization experiments, every cell line was freshly grown in MTAP medium. The cells were harvested by centrifugation until they reached their log phase of growth (~1.5×10^6 cells mL^{-1}). To verify the expression and localization of the chimeric PM protein, the fluorescence emitted from the cells was first quantified using a BD Accuri™ C6 Plus flow cytometer (BD Bioscience, USA) using BD Accuri™ C6 Plus software (BD Bioscience, USA). The harvested cell pellet was resuspended in freshly prepared medium. The flow was continued until 1×10^4 single-cell events were detected, which resulted in a total of 3×10^3 events tightly gated based on the forward (FSC-A) *vs*. and side scatter (SSC-A) profiles that covered the most densely populated region of each strain (Supplementary Figs. 9a–d and Supplementary Table 3). To statistically analyze the distribution of the cell population according to the fluorescence intensity, smoothed histograms were plotted with FlowJo™ software (BD Bioscience, USA). For the cytometry analysis, the cells were excited with a 488 nm laser and the emission was detected using a 533/30 nm bandpass filter (FITC-A).

To verify the subcellular localization, confocal fluorescence microscopy was performed. The cell pellet corresponding to 1.5×10^7 cells (*i.e.*, 10-fold concentrated) was resuspended in 1 mL of fresh cell medium. Five microliters of the cell suspension was placed onto a microscope slide glass and blended with an equivalent volume of MTAP medium-based low melting temperature agarose (Sigma-Aldrich, USA) solution (1% w/v) to immobilize the algal cells. The exact localization of the exogenously expressed protein was visually identified based on the fluorescence emitted from the fluorescent tag (*i.e.,* mVenus). Using a Zeiss LSM 800 confocal laser scanning microscopy system (Zeiss, Germany), the fluorescent probe was excited with a 528 nm argon ion laser, and the emission was detected employing a 535/590 nm bandpass filter. Moreover, the autofluorescence from chloroplasts was visualized with a 655 nm excitation laser and 656/700 nm bandpass emission filter. The contrast and brightness of the resulting images were identically processed using ZEN software (Zeiss, Germany) to improve the visibility.

ELISA was performed to detect GFP derivatives with the PM-enriched fraction to further specify the localization of the protein. First, the subfraction was isolated from the algal cells according to a previous study

with several modifications.[6] In detail, the algae were cultured in 200 mL of MTAP medium and harvested when the cell density reached ~3 × 10^6 cells mL^{-1}. The collected cell pellet was then resuspended in homogenization buffer, which consisted of 0.5 M sorbitol, 50 mM Tris-HCl, pH 7.5, 5 mM Na_2EDTA, 5 mM dithiothreitol, 1 mM phenylmethylsulfonylfluoride, and 2.5% polyvinylpyrrolidone. The algal cells were then disrupted by ultrasound treatment for 5 min using a Sonics® Vibra Cell VC 130 ultrasonic processor. Sonication was conducted with a power output of 30 W and a pulse duration of 10 s. All the procedures were performed in an ice bath. Proper fragmentation was confirmed by microscopy. The lysate was centrifuged using a prechilled (at 4 °C) Himac CF 16RN centrifuge (Hitachi, Japan) at 14,000 xg for 30 min to remove cell debris. The soluble protein-containing supernatant was centrifuged again with a Himac CP100 WX ultracentrifuge (Hitachi, Japan) at 100,000 xg for 30 min to pelletize the desired subcellular component. The obtained pellet was resuspended in homogenization buffer. Subsequently, the PM-enriched fraction included in the homogenate was further refined using the dextran/PEG aqueous two-phase partitioning system. Namely, the suspended fraction was mixed with 6.4% (w/w) dextran T500, 6.4% (w/w) PEG 3350, and 8 mM NaCl (all representing the working concentration) and then subjected to gentle centrifugation at 1,000 xg for 5 min. The PM-containing upper phase was centrifuged again at 100,000 xg for 60 min. After removing the supernatant, the precipitate (*i.e.*, PM-enriched fraction) was finally resuspended in 100 µL of a protein lysis buffer consisting of 50 mM Tris-HCl and 100 mM NaCl (pH 7.0). Following the separation step, the PM-enriched sample was subjected to ELISA to verify the existence of fluorescent probe-fused PMA in the PM-enriched fraction (corresponding to 9 µg of PM protein). According to the manufacturer's protocol, the immunoassay was performed in duplicate using an AKR-121 GFP ELISA Kit (Cell Biolabs, Inc. USA), which is capable of detecting a wide range of GFP derivatives, and the resulting spectrophotometric data were acquired using an Infinite® 200 PRO microplate reader (Tecan, Switzerland). All reagents used in this step were obtained from Sigma-Aldrich (USA).

Supplementary Method 6. Gross photosynthesis rate

The algal cells were freshly grown in MTP autotrophic medium under atmospheric air conditions with a continuous light illumination of 50 µE m^{-2} s^{-1} at 23 °C. For the air-grown cell group, the cells were repeatedly subcultured under identical conditions. For the high CO_2-treated group, the algal strains were suddenly transferred to the highly CO_2-enriched condition (air-balanced 20% CO_2) one day prior to the measurement without acclimation. After 3 days of cultivation, as the cells neared their exponential phases (both air- and high CO_2-grown groups), they were harvested by centrifugation, resuspended in correspondingly equilibrated autotrophic media

and then separately incubated under the two different CO_2 conditions with vigorous shaking, and the rate of change in the dissolved O_2 concentration was then monitored. During resuspension, the cell numbers of each strain were adjusted to 1.5×10^6 cells mL^{-1}. The O_2 evolution and dark respiration rates were then quantified using the Oxygraph Plus oxygen electrode system (Hansatech, UK). The surrounding temperature of the cell chamber was maintained isothermally at 23 °C using a recirculating water bath. The per-cell gross photosynthetic rate of the algal strains (in nmol 10^6 cells^{-1} min^{-1}) was estimated based on the addition of 10 mM $NaHCO_3$ (final concentration, as an inorganic carbon source for oxygenic photosynthesis) by summation of the net photosynthetic oxygen evolution rate at 50 μE m^{-2} s^{-1} (equal to the growth condition) and dark respiration rate.[7] The respiration rate was evaluated with the O_2 consumption rate in the absence of light illumination.

Supplementary Method 7. Intracellular pH measurement

A pH-sensitive, cell-permeable fluorescent indicator, BCECF-AM (Molecular Probes, USA), was employed to investigate the effect of high CO_2 conditions on pH_i acidification. The nonionic surfactant Pluronic™ F-127 (Invitrogen, USA) was used to promote the endocytosis of the dye.[8] Both the dye and surfactant were dissolved in DMSO (Sigma-Aldrich, USA) in advance. The pH_i was then ratiometrically measured according to the following process. The algal cells were maintained autotrophically under ambient conditions. Shortly before the pH_i test, the cells were harvested and resuspended in modified PBS, which exhibited improved buffer capacity *via* supplementation with additional buffer materials, such as MES and Tris base. The modified PBS consisted of 10 mM Tris, 10 mM MES, 137 mM NaCl, 2.7 mM KCl, 4.3 mM Na_2HPO_4, and 1.4 mM KH_2PO_4 and was adjusted to pH 7.0.[9] Following adjustment of the cell number to approximately 5×10^6 cells mL^{-1}, the cell suspensions were loaded with 10 μM BCECF-AM and 0.0025% (w/v) Pluronic™ F-127. The sample was incubated for 1 h at 23 °C under atmospheric conditions with 50 μE m^{-2} s^{-1} light illumination to offer ample time for the uptake of nonfluorescent BCECF-AM into the cytosol and the intracellular cleavage of AM ester bonds by nonspecific esterases to yield a fluorescent BCECF-free acid.[10] Subsequently, the dye-treated cells were harvested by applying centrifugal force at 1,373 xg for 5 min. The collected cell precipitates from the air-grown and high CO_2-treated groups were resuspended in air-saturated and 20% CO_2-saturated modified PBS buffer solutions without the dye and surfactant, which were prepared by shaking vigorously under the designated conditions beforehand. Washing steps were carried out with buffer solutions where appropriate. The cell groups were immediately placed in two CO_2 conditions. For the air-grown group, the cell lines were placed under the same ambient conditions at which they were previously grown, whereas for the high CO_2-treated group, the algal strains were transferred to

extremely high CO_2 conditions (*i.e.*, air-balanced 20% CO_2). Both cell groups were incubated with continuous light illumination (50 μE m^{-2} s^{-1}) at 23 °C with vigorous shaking (120 rpm). To observe the time-dependent changes in the cytosolic pH, each group was exposed to the respective CO_2 conditions for 1 h and 2 h independently and 1 mL aliquots of the cell suspensions were taken and washed with the corresponding modified PBS solutions. The resuspended cell sample was used for pH_i measurements. The dual excitation dye in the intracellular region was excited at both 490 nm and 439 nm, and the emissions were identically detected at 535 nm using an Infinite® 200 PRO microplate reader system (Tecan, Switzerland). The fluorescence ratio (R, 490/439) was then determined with the background-corrected emission intensities, whereas the background fluorescence values were measured for the dye-unloaded cells.[11]

To convert the R values into pH_i values, an *in situ* pH calibration was performed. The dye loading process proceeded as described above. Instead of being washed with air- and CO_2-saturated buffers, the cells were separately resuspended in modified PBS buffer solutions with 5 different pH values (5.0, 6.0, 7.0, 8.0, and 9.0) in the absence of dye and surfactant supplementation. The pH was titrated with HCl and KOH. The cells were washed twice with pH-adjusted PBS solutions and incubated for 1 h and 2 h independently. Thirty minutes prior to the end of each incubation, the algal cells were supplied with high [K^+] (final working concentration of 135 mM) and 30 μM ionophore cocktail consisting of 10 μM nigericin, 10 μM monensin, and 10 μM carbonyl cyanide-p-trifluoro methoxyphenyl hydrazone to equalize the pH_i and external pH by rapidly collapsing the cell membrane potential.[11,12] The background-corrected R values were calculated and subsequently fitted with the 4-parameter logistic (sigmoidal) function using SigmaPlot (Systat Software, USA) software (Supplementary Fig. 13).[13]

Supplementary Method 8. *In vitro* ATPase activity test

The *in vitro* ATPase activity was monitored using the PM-enriched fractions (same samples used for analysis of the protein localization by ELISA) with the same amount of protein acquired from each strain based on the release rate of inorganic phosphate (P_i) *via* ATP hydrolysis.[14] ATP hydrolysis was processed in 100 μL of reaction solution consisting of 4 mM Mg ATP, 1 mM free Mg^{2+} ($MgCl_2$), 10 mM sodium azide (as a mitochondrial ATPase inhibitor), 0.2 mM molybdate (as a protein phosphatase inhibitor), 20 mM KNO_3 (as a vacuolar ATPase inhibitor), 0.05% Brij 58, 25 mM MES, 25 mM HEPES and 8 μg of PM proteins at 30 °C for 40 min. Subsequently, 500 μL of 1% SDS solution was added to the mixture to end the hydrolytic reaction. To spectroscopically determine the pH_i concentration, color development was initiated with the addition of 400 μL of the reagent, which was prepared

with of 2% (w/v) ascorbic acid and 40% (v/v) molybdate reagent solution (Sigma-Aldrich, USA), and continued by subsequent incubation at 45 °C for 45 min. Following incubation, the absorbance (OD_{700}) of the sample was measured using a UV-1800 UV spectrophotometer (Shimadzu, Japan). The final data are shown as baseline-subtracted rates. To determine the baseline of the reaction, control experiments without the PM-enriched fraction were performed for the quantification of unintended ATP autolysis. Sequentially diluted K_2HPO_4 solutions were used as standard materials for calibrating the P_i concentration of the solution.

Supplementary Method 9. *In vivo* ATPase activity test

To estimate the H^+ extrusion performance of the microalgal strains at the whole-cell level, a colorimetric pH indicator, bromocresol purple (5',5''-dibromo-o-cresolsulfophthalein), was adopted. This indicator enables colorimetric determination of the pericellular acidification on the back of the microalgal H^+ discharge by a change in the cell medium's color from brown to light yellow.[15] Freshly grown algal cells in liquid mixotrophic MTAP medium were collected by centrifugation, and the cell pellet was resuspended in MTAP medium with no buffer materials (*i.e.,* Tris and MES) and an adjusted pH of 6.0. During resuspension, the cell density was normalized to 1.5×10^6 cells mL^{-1}. Using the same medium (at pH 6.0), a solid agar plate consisting of 1.5% (w/v) plant agar and 0.005% bromocresol purple was prepared. On solid medium, 20 μL of the cell suspensions containing the same number of algal cells were spotted and incubated for 3 days with light illumination (50 $\mu E\ m^{-2}\ s^{-1}$) at 23 °C. A slightly acidic pH condition was set up to facilitate discrimination of the difference in the cellular H^+ extruding ability.

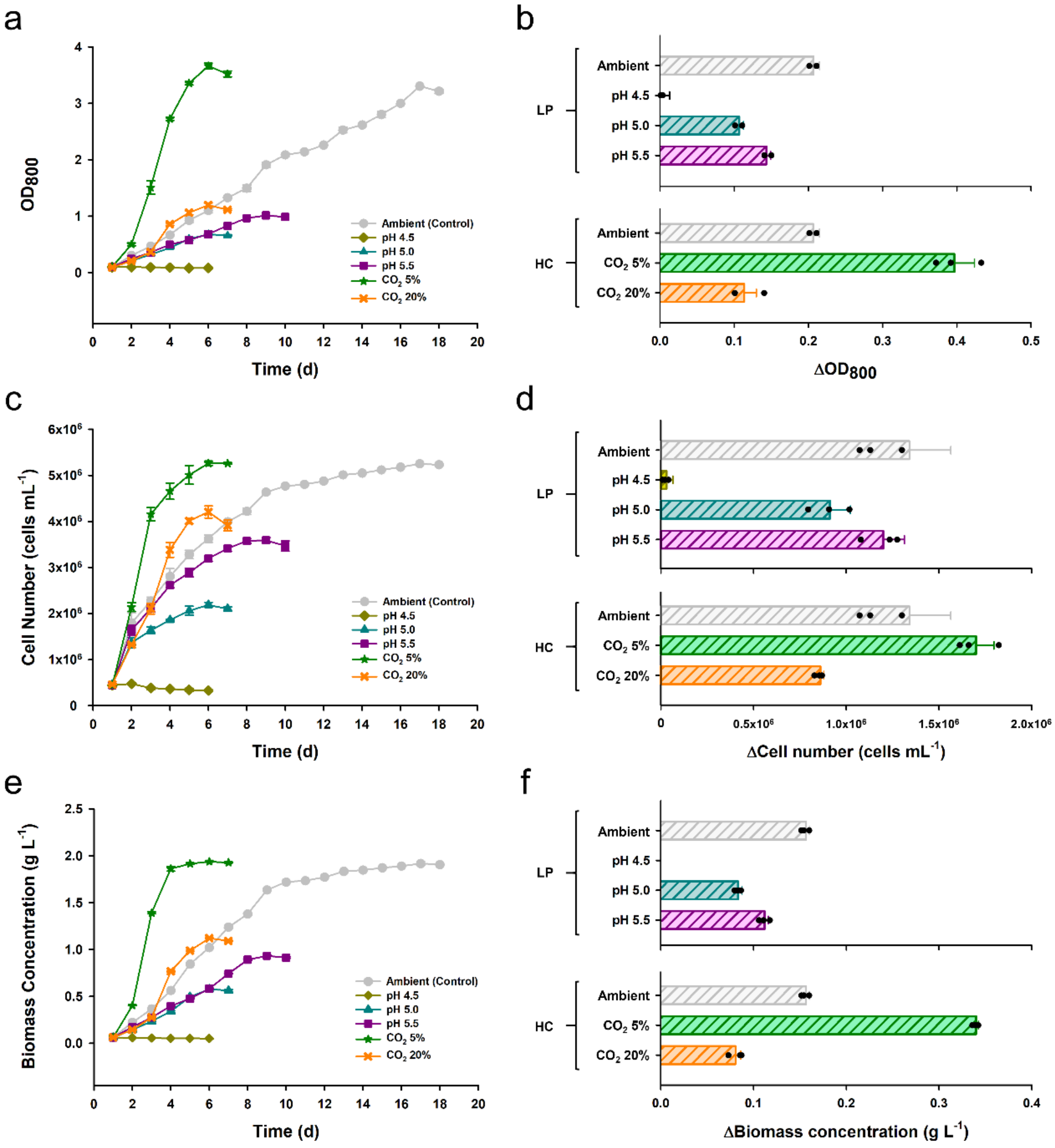

Supplementary Figure 1. Growth behavior of *Chlamydomonas reinhardtii* under various pH (pH 4.5, pH 5.0, and pH 5.5) and CO_2 (5% CO_2 and 20% CO_2) conditions (n = 3) in terms of (a) daily turbidity (OD_{800}), (c) cell number, and (e) biomass concentration. Supplementary Figs. 1b, 1d, and 1f show the changes in (b) the optical density, (d) cell number, and (f) biomass concentration during the first incubation day, respectively. The correlation between ΔOD_{800} (b) and Δbiomass concentration (f) clearly shows that the optical density represents the biomass concentration of the culture. EC_{50} was determined by comparing the biomass concentration difference under specific pH and CO_2 conditions with that under ambient control (pH 7.0 atmospheric state) conditions. In Supplementary Figs. 1b, 1d and 1f, LP and HC denote low pH and high CO_2 conditions, respectively, and each of the paired graphs shares the same lateral axis. Shown are data from triplicate cell cultures. Data are the mean ± SD of three biological replicates. Source data are provided as a Source Data file.

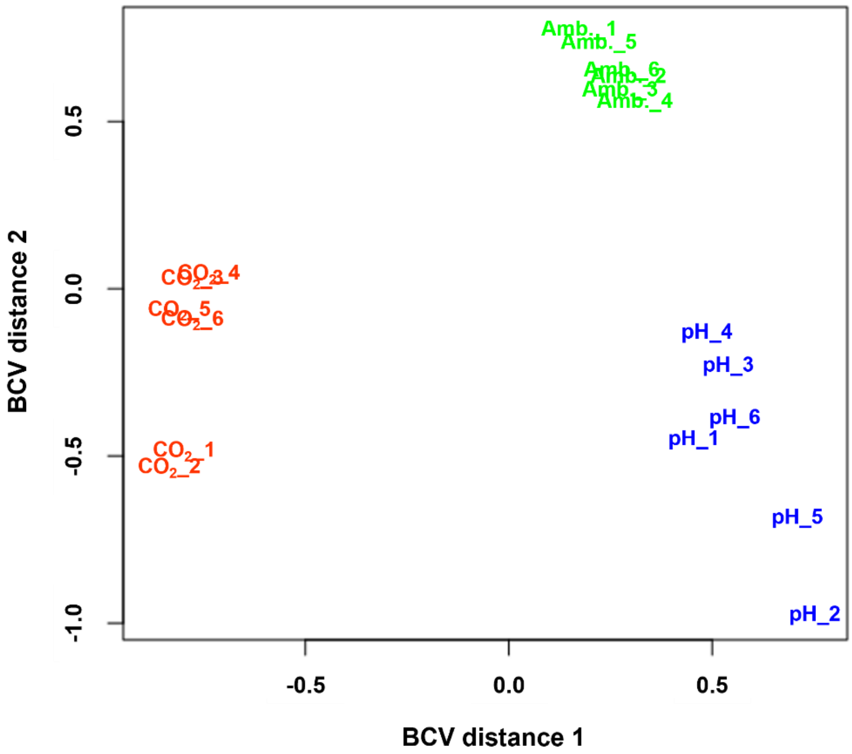

Supplementary Figure 2. Multidimensional scaling (MDS) plot that shows the measured similarity of the RNA-seq samples. The distances between two points reflect the leading biological coefficient of variation (BCV) of the corresponding RNA-seq samples. Amb., pH, and CO_2 (1 to 6) represent six biological replicates (n=6) of the ambient (atmospheric CO_2), low pH (pH 5.0), and high CO_2 (CO_2 20%) conditions, respectively.

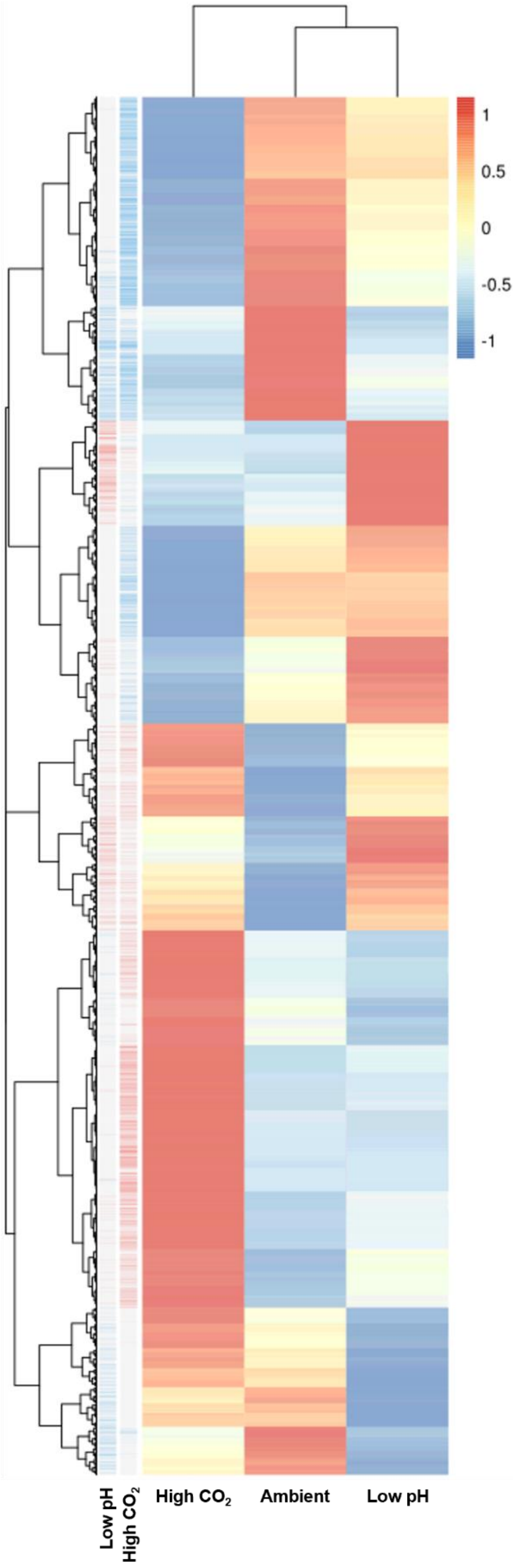

Supplementary Figure 3. Heatmap of the global transcriptome profile of *C. reinhardtii* WT cells grown under ambient, low pH (pH 5.0), and extremely high CO_2 conditions (air-balanced 20% CO_2).

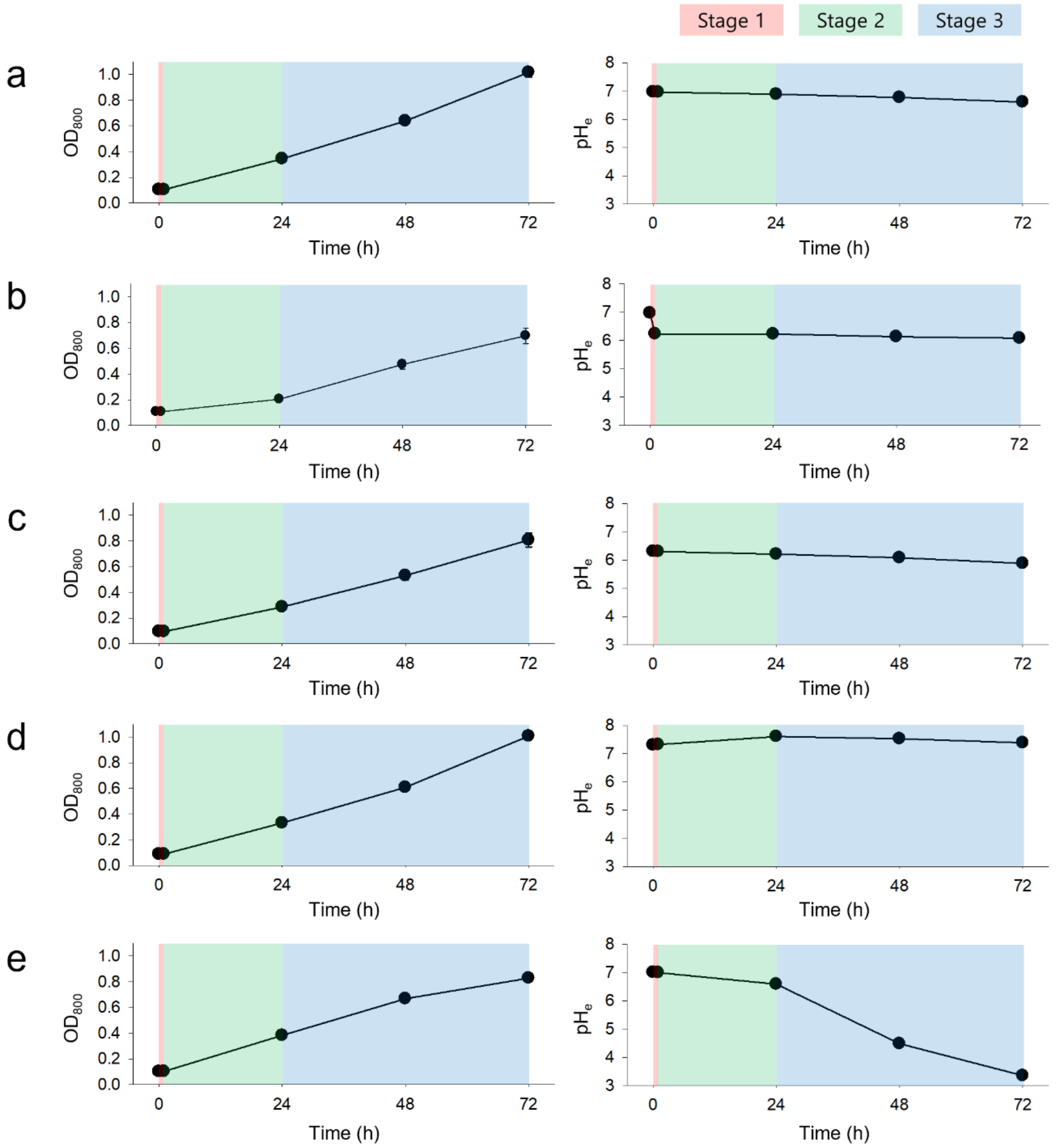

Supplementary Figure 4. Analysis of the inhibitory effects of high CO_2 conditions on *C. reinhardtii* WT strain CC-125. Data are the mean ± SD of two biological replicates. Growth and extracellular pH (pH_e) profiles during culture of the cells under (a) ambient, (b) high CO_2 (20% CO_2), (c) CO_2-independent low pH (pH 6.2), (d) CO_2-independent high [DIC] (7 mM), and (e) nonbuffered ambient conditions. (e) was investigated as a control to demonstrate the medium acidification effect of ammonium uptake (as a nitrogen source). The cultivation period was divided into three stages in reference to the external pH behavior (Stage 1: from 0 h to 1 h; Stage 2: from 1 h to 24 h; and Stage 3: after 24 h). Source data are provided as a Source Data file.

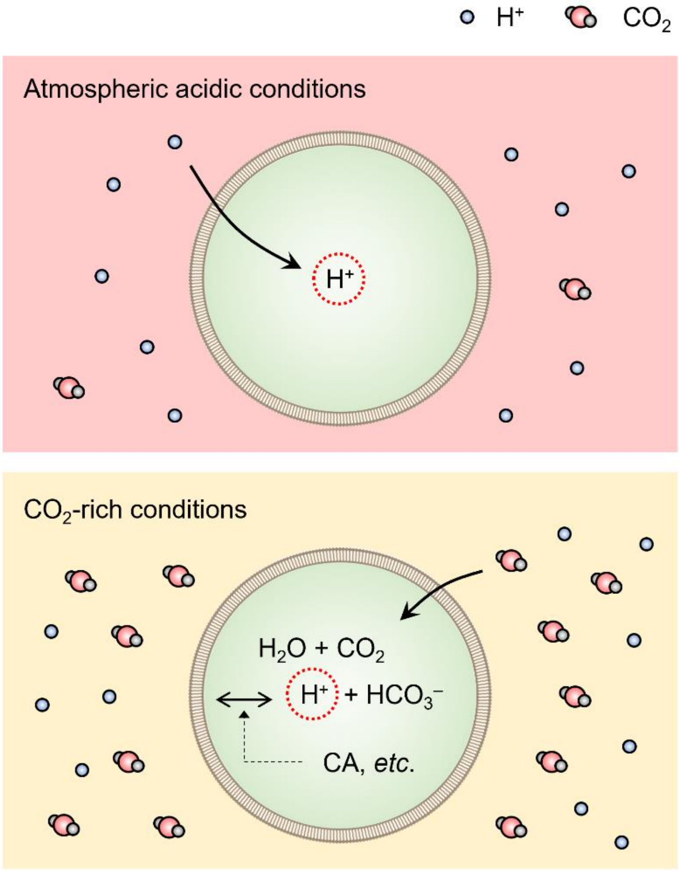

Supplementary Figure 5. Schematic diagram demonstrating the aggravation of the intracellular acidification process under high CO_2 conditions. Under CO_2-independent acidic conditions (*i.e.* atmospheric acidic conditions), intracellular acidification occurs solely due to H^+ in the surroundings. In addition, under high CO_2 conditions (*i.e.* CO_2-rich conditions), a decrease in the pH_i would be exacerbated through the facilitation of intracellular CO_2 hydration (and concomitant H^+ generation) in addition to the effect of extracellular acidity. In the schematic diagram, CA represents carbonic anhydrase. The relative number of CO_2 in the upper and lower figures was determined in reference to a previous study.[1]

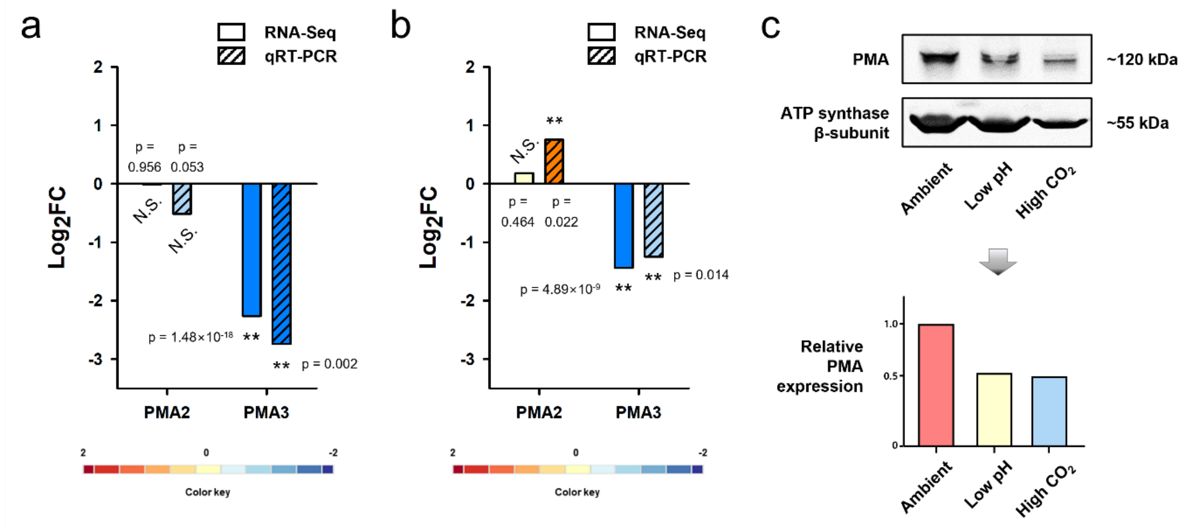

Supplementary Figure 6. qRT-PCR validation (n=3 biologically independent samples) of the transcriptome analysis results (n=6 biologically independent samples) from (a) high CO_2 conditions and (b) CO_2-independent low pH conditions. The double asterisks in the plot indicate statistically significant differences in the expression levels ($p < 0.05$) under the corresponding culture conditions compared with those found under ambient conditions. N.S. represents not significant. In (a) and (b), the statistically significant differences for the qRT-PCR results were estimated based on a two-tailed Student's t test, while that for the transcriptome analysis were estimated based the generalized linear model (GLM) likelihood ratio (LR) test. (c) The downregulation of PMA expression at the protein level under low pH and high CO_2 conditions was confirmed by the Western blotting band intensity observed from 200 μg of protein loading (see Supplementary Method 4 for details). This result implies the mRNA-protein expression correspondence and, at the same time, suggests that the downregulation can directly influence the intolerance. The bands were observed at approximately 120 kDa which is the expected molecular weight of the algal native PMA which is estimated from its amino acid sequence (the expected molecular weight of the algal PMA: 117.89 kDa). The expression of AtpB (ATP synthase β-subunit; ~55 kDa) was observed as the loading control for each condition. Using the band image, the relative PMA expression level was quantified based on the major band intensities using ImageJ software. Source data are provided as a Source Data file.

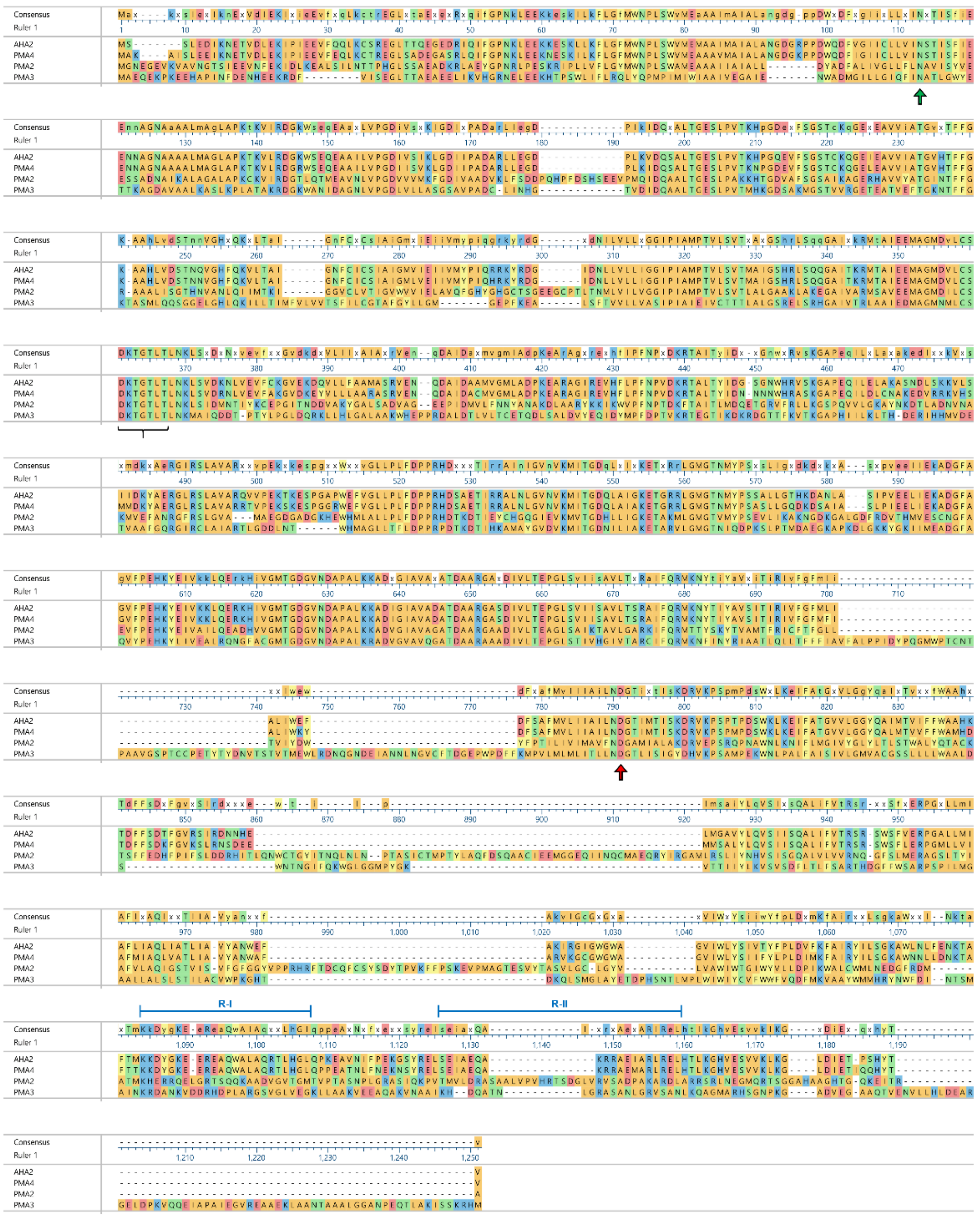

Supplementary Figure 7. The sequence alignment of PMA2 and PMA3 (*C. reinhardtii* plasma membrane H^+-ATPases) with AHA2 (autoinhibited PM H^+-ATPase 2, *Arabidopsis thaliana* plasma membrane H^+-ATPase) and PMA4 (*Nicotiana plumbaginifolia* plasma membrane H^+-ATPase). The latter both are well characterized plant PMAs. The alignment was conducted with DNASTAR MegAlign Pro™ software using the MAFFT method. Asn106 (N106) and Asp684 (D684), all of which play a crucial role in H^+ transport, are indicated with green and red arrows, respectively, and a highly conserved region (DKTGTLT) of P-type ATPases is designated with a black bracket. R-I and R-II denote regions I and II, respectively, which are highly conserved sequences that appear in the autoinhibitory domain of terrestrial plant PMAs (*e.g.*, AHA2 and PMA4). The absence of a penultimate threonine-containing sequence at the C-terminal regions (*i.e.*, His/Ser-Tyr-pThr (phosphorylated Thr)-Val-COOH), unlike plant PMAs, implies that the activities of algal PMAs (*i.e.*, PMA2 and PMA3) are not regulated by general regulatory 14-3-3 proteins.[16]

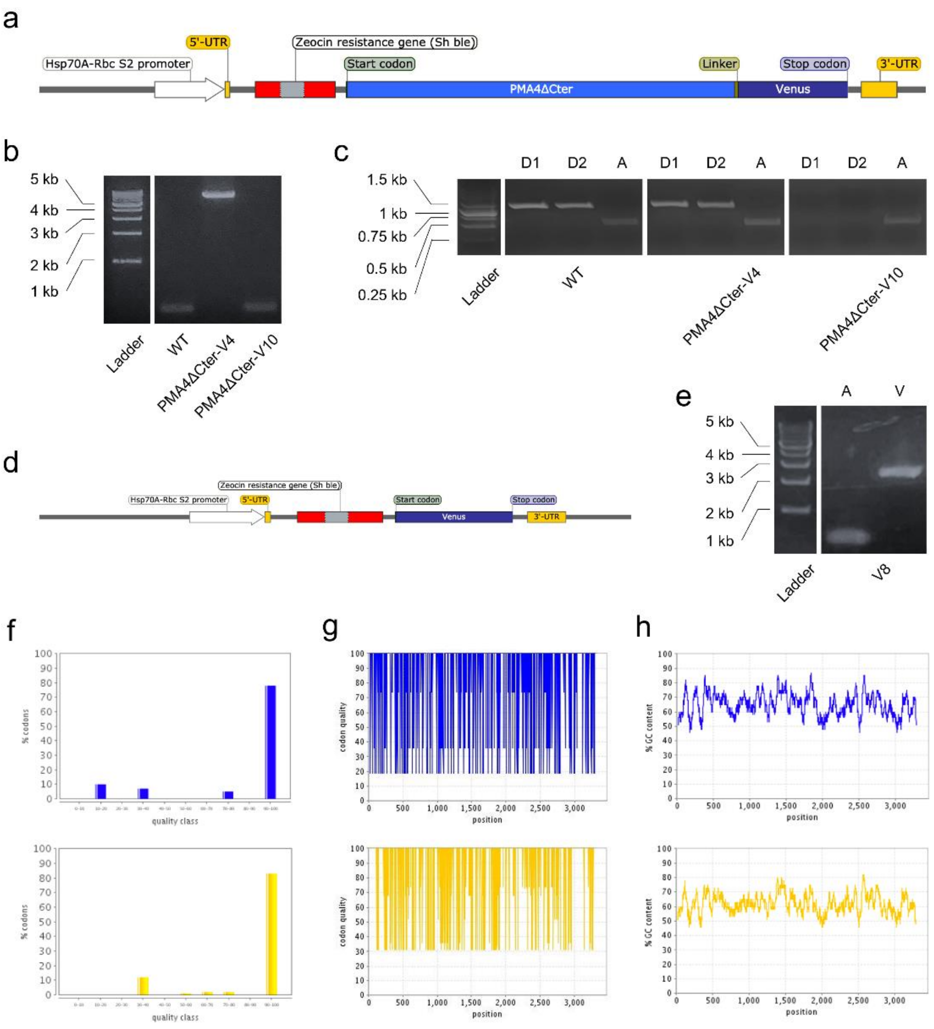

Supplementary Figure 8. Linearized insert cassettes for expressing (a) PMAΔCter-V (6835 bp) and (d) mVenus (4258 bp) generated using SnapGene® software. The zeocin resistance gene (*Sh* Ble) coding sequence includes an intron region (indicated with a gray box) that is supposed to be removed from the mature mRNA through the splicing process. PCR confirmation of the insertion site in (b) PMA4ΔCter-V4 and (c) PMA4ΔCter-V10 using appropriate primers (Supplementary Table 1). For the PMA4ΔCter-V4 strain, extension of the amplicon as a result of gene insertion was observed. On the one hand, because a wide range of genomic DNA sequences was lost in the PMAΔCter-V10 strain (5461 bps; revealed by NGS) and the length of the lost gene is similar to that of the insert cassette, it is difficult to distinguish whether the insertion-derived extension occurred based on a general electrophoresis method. Given this, deletion of two different gene sequences (1198 and 1136 bps for D1 and D2, respectively), which are interbedded in the lost sequence, was verified to alternatively confirm the gene insertion. The absence of PCR bands in the PMAΔCter-V10 mutant is indicative of the gene deletion. D1, D2, and A represent the deleted sequences 1, 2, and the actin gene (an endogenous control), respectively. (e) PCR-based genotyping of only mVenus expressing the V8 mutant. A and V indicate the actin (an endogenous control) gene and the mVenus insert gene (expected amplicon length: 2114 bp), respectively. (f)–(h). Codon optimization of PMAΔCter-V according to codon usage in the nuclear genome of *Chlamydomonas reinhardtii* before (left panel) and after (right panel) optimization. The plots show (f) the percentage of codons that fall into a certain quality class, (g) the quality of the used codon at the indicated position, and (h) the GC content by focusing on a 40-bp window. Using an identical approach, the gene sequence encoding mVenus was also codon-optimized. Source data are provided as a Source Data file.

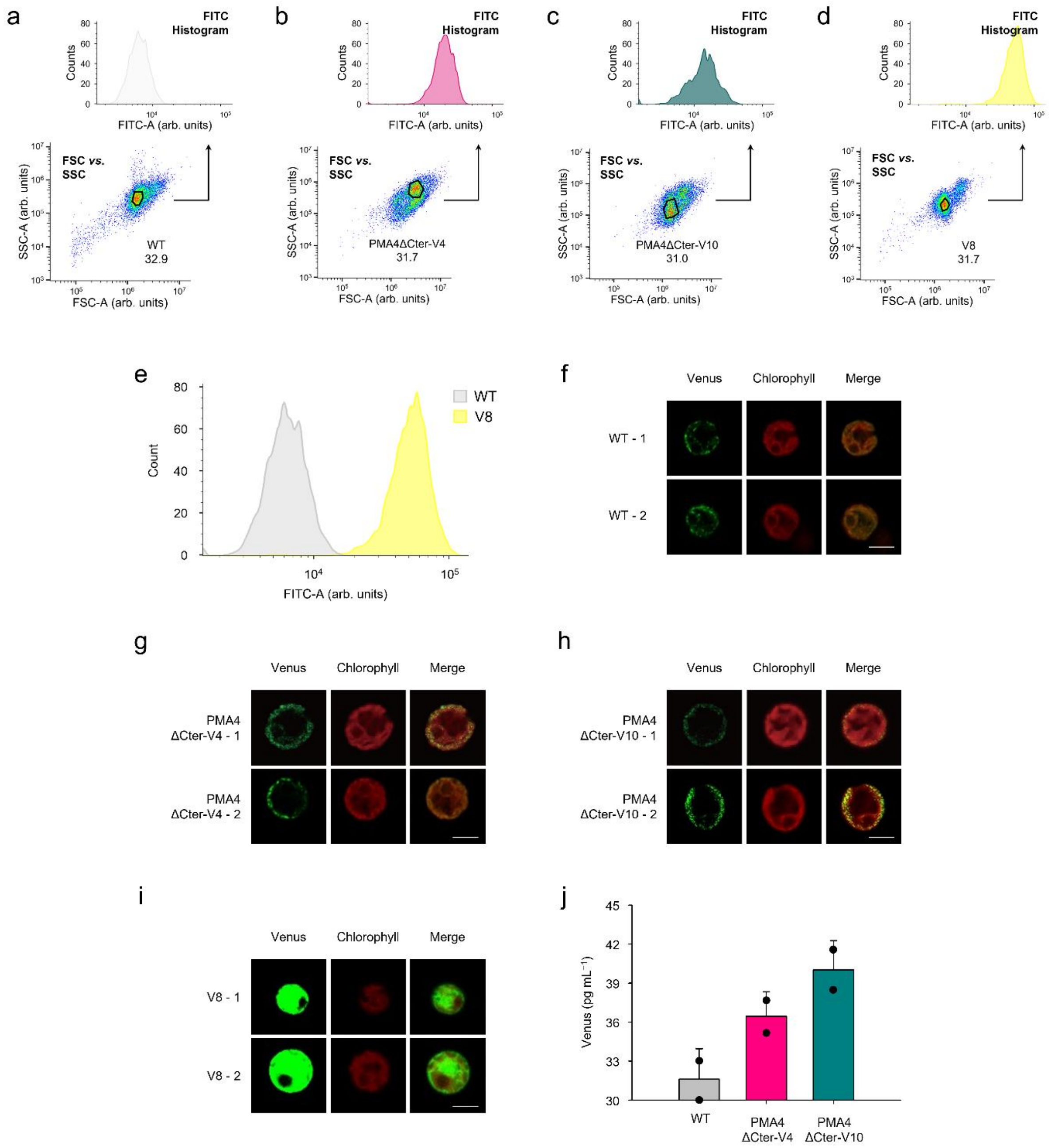

Supplementary Figure 9. Probing the subcellular localization of the expressed protein. Gating strategy for detection of the cellular fluorescence of (a) WT, (b) PMAΔCter-V4, (c) PMAΔCter-V10, and (d) V8 cells. In Supplementary Figs. 9a–d, each gate in the FSC *vs*. SSC plots, covering a similar number of events (approximately 3×10^3 events) from the most densely populated region, was used to estimate the fluorescence (FITC-A) of each cell line (see Supplementary Method 5 for details). (e) Results of the flow cytometry analysis (WT and V8). Confocal fluorescence images of (f) WT cells, (g) PMAΔCter-V4 cells, (h) PMAΔCter-V10 cells, and (i) V8 cells. Scale bar = 5 µm. (j) Detection of mVenus by enzyme-linked immune sorbent assay (ELISA; Cell Biolabs, Inc. USA). In the assay, the plasma membrane enriched fraction separated by a two-phase partitioning system was used. The axis of the ordinates starts from the lower limit of the linear detection range (*i.e.*, 30 pg mL^{-1}) indicated by the manufacturer (n=2 biologically independent samples). Source data are provided as a Source Data file.

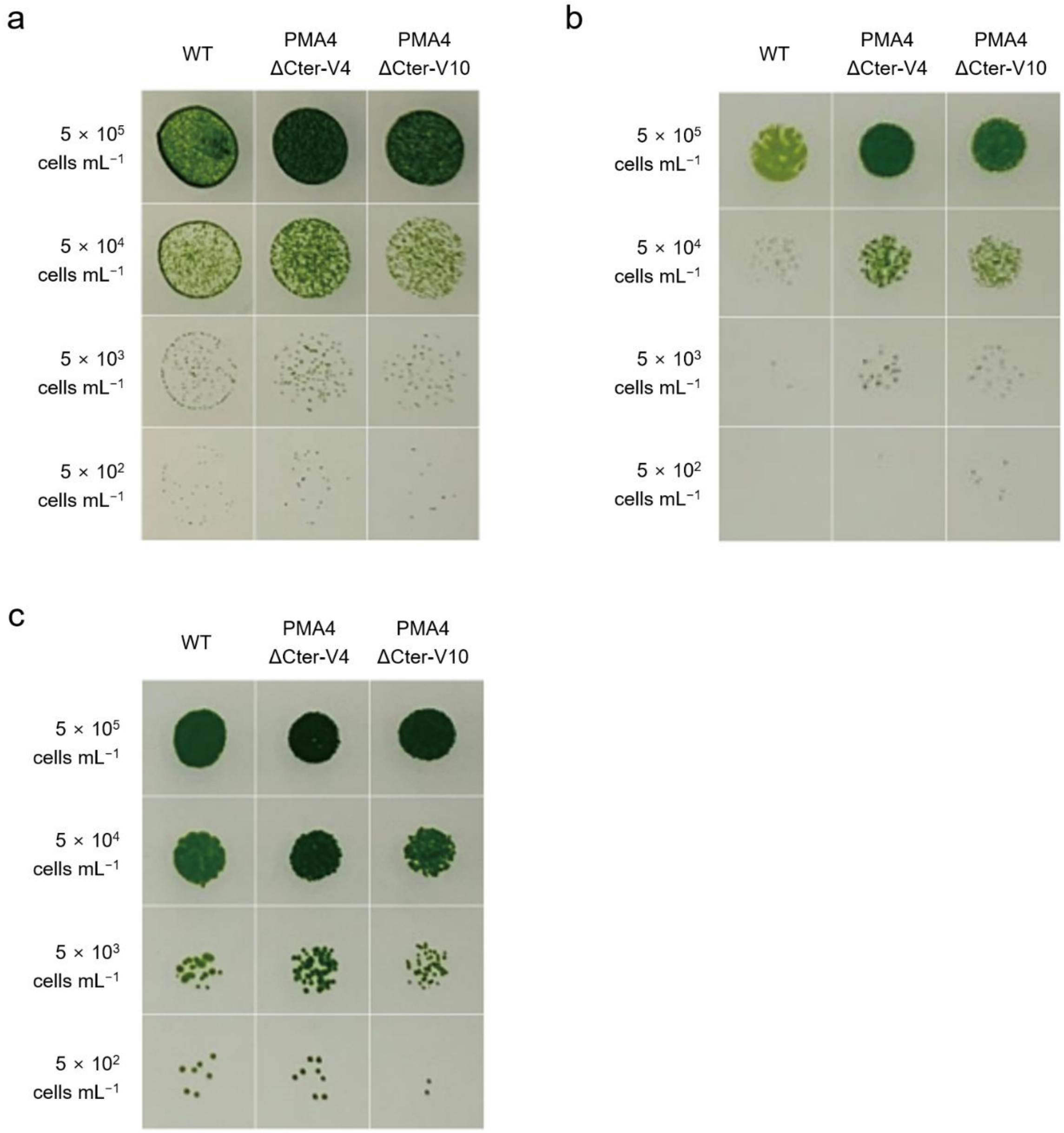

Supplementary Figure 10. Solid spot culture results. Serial dilution culture of (a) mixotrophic cultivation (supplemented with acetic acid) at neutral pH (pH value of 7.0), (b) autotrophic cultivation at a low pH (pH value of 5.5), and (c) autotrophic cultivation at neutral pH (pH value of 7.0). Source data are provided as a Source Data file.

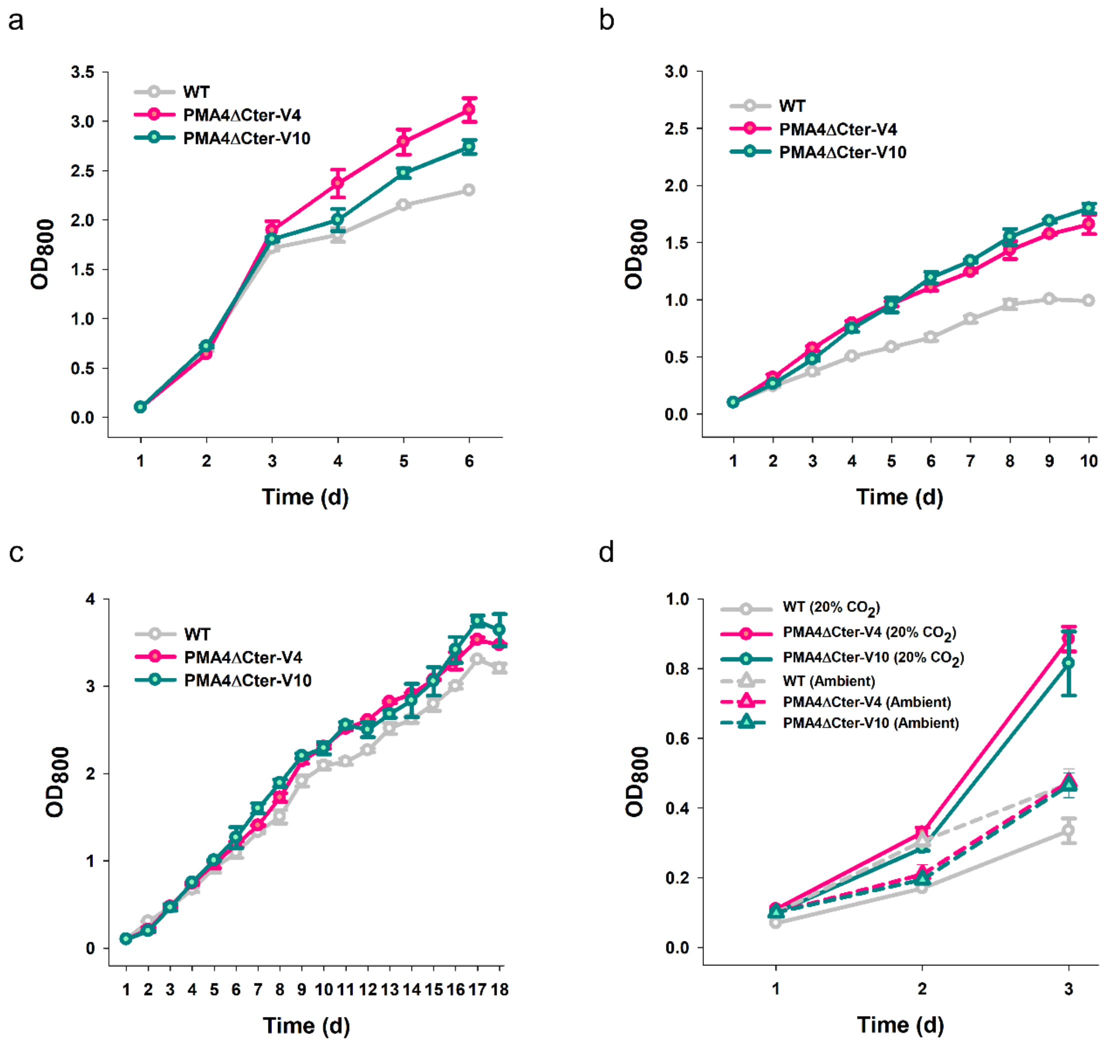

Supplementary Figure 11. Growth curves from the liquid cultivation of (a) mixotrophic cultivation at neutral pH (pH value of 7.0), (b) autotrophic cultivation at a low pH (pH value of 5.5), and (c) autotrophic cultivation at neutral pH (pH value of 7.0). The growth performances were observed daily until the cell lines reached their maximum concentrations. (d) Compiled photoautotrophic growth data obtained for the algal strains (focusing on their initial growth stages; between Day 1 and 3) grown under two different CO_2 conditions (*i.e.*, ambient CO_2 and 20% CO_2 conditions). Data from duplicate cell cultures of each strain are shown. Source data are provided as a Source Data file.

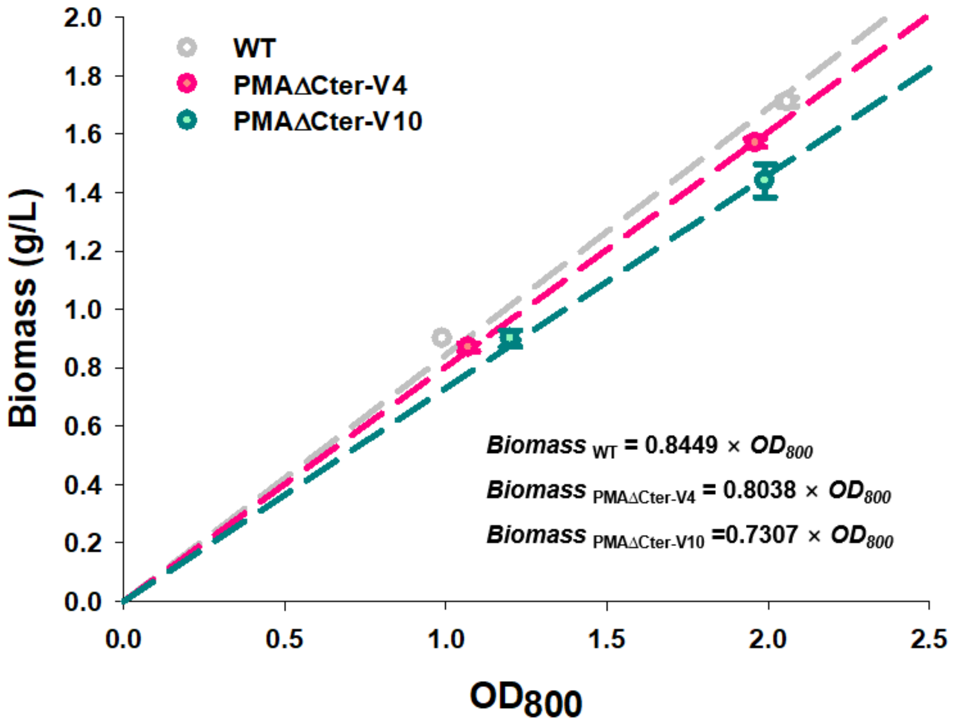

Supplementary Figure 12. Correlation between the biomass concentration (in g L^{-1}) and OD_{800} for each cell line under ambient photoautotrophic conditions. Data are the mean ± SD of two biological replicates. Source data are provided as a Source Data file.

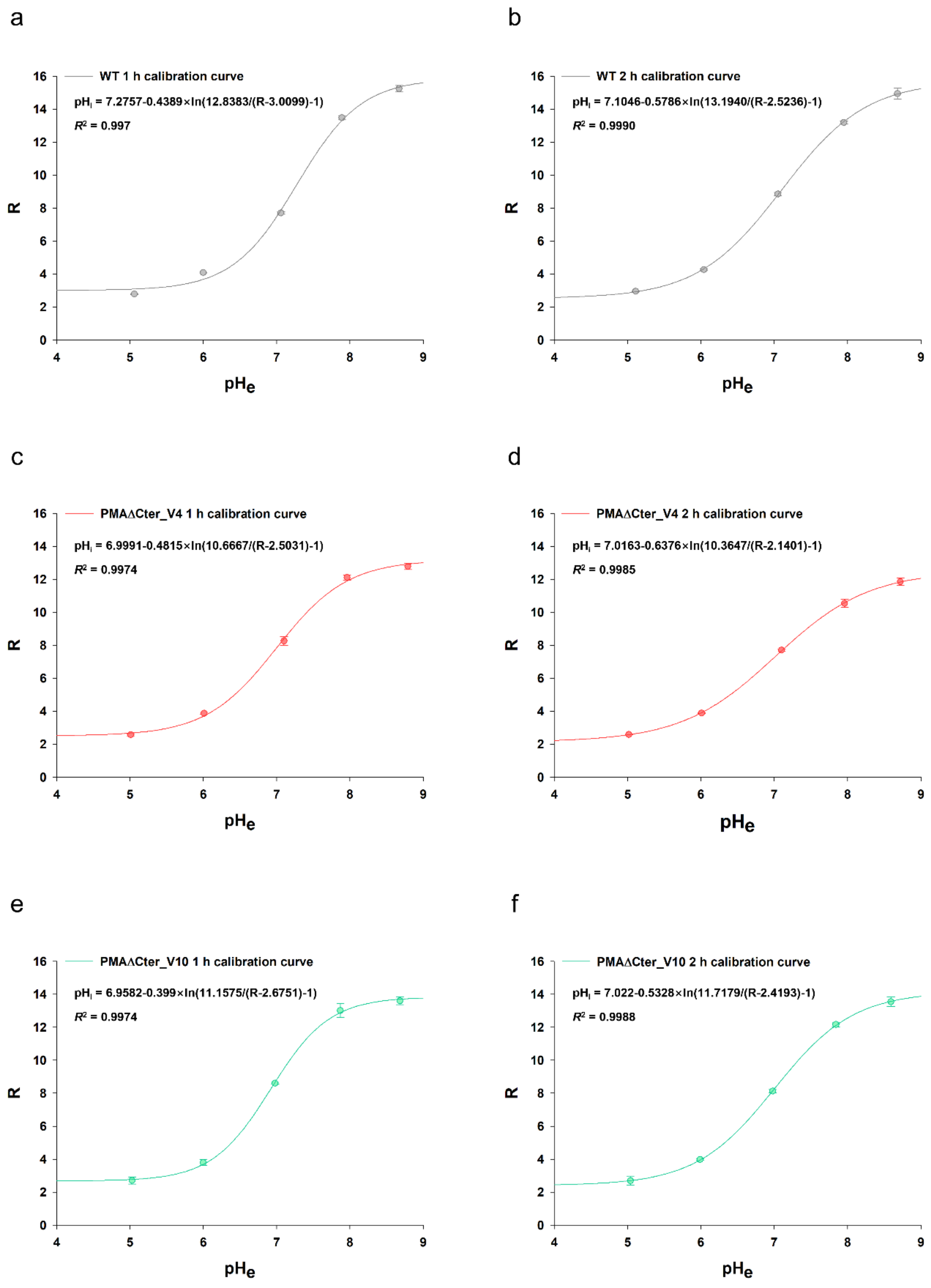

Supplementary Figure 13. pH_i calibration curves for each strain (n = 4): (a) WT 1 h, (b) WT 2 h, (c) PMAΔCter-V4 1 h, (d) PMAΔCter-V4 2 h, (e) PMAΔCter-V10 1 h, and (f) PMAΔCter-V10 2 h. Data are the mean ± SD of four biological replicates. Source data are provided as a Source Data file.

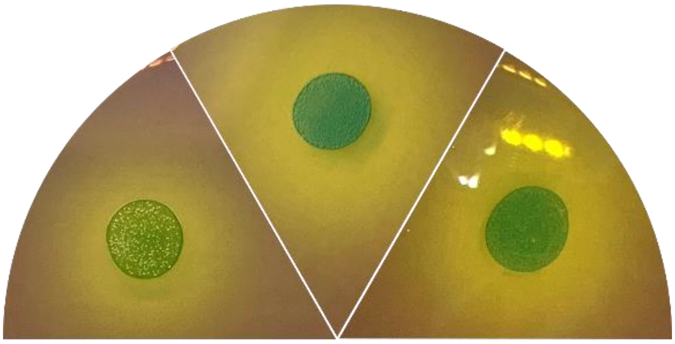

Supplementary Figure 14. Bromocresol purple-mediated assessment of *in vivo* ATPase activity in duplicate (with Fig. 3g). Source data are provided as a Source Data file.

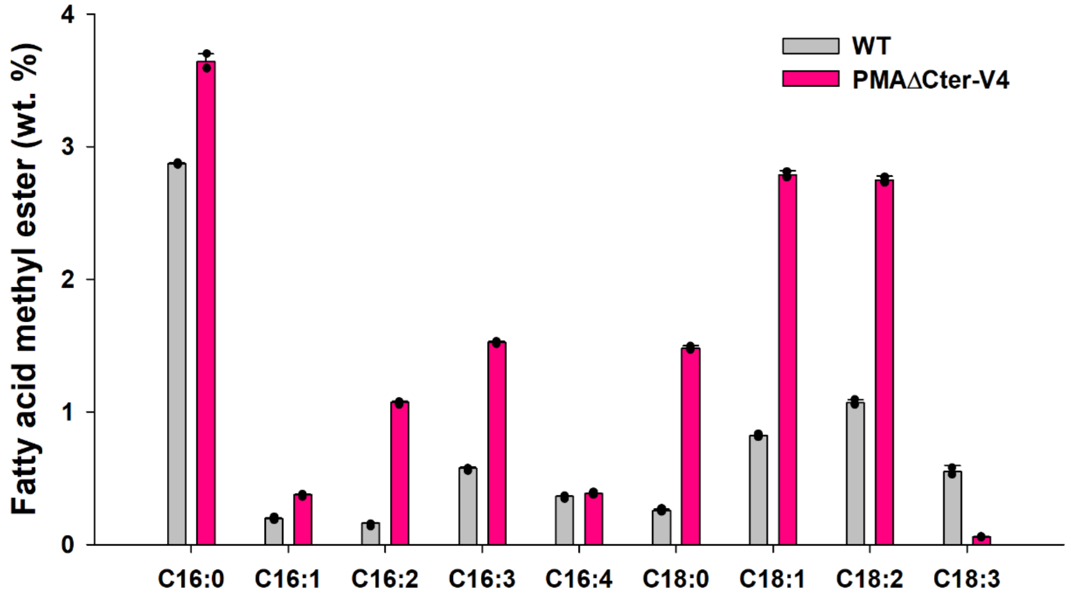

Supplementary Figure 15. Profile of fatty acid methyl esters (FAMEs) of WT and PMAΔCter-V4 subjected to outdoor cultivation using coal-fired flue gas. Data are the mean ± SD of two biological replicates. Source data are provided as a Source Data file.

Supplementary Table 1. Primer sequences for colony PCR and qRT-PCR in this study.

Set	Amplifying target	Primer sequence	Amplicon size (bp)	Reference
Colony PCR	IDA5 (coding an actin protein; as an endogenous control)	Forward: 5'-TCGTCACCTTATCCTCATCGC-3'<br>Reverse: 5'-CTGCAAGCTATCCACGTATGC-3'	501	5
	PMA4ΔCter-V	Forward: 5'- AGCTACAGGACTGATTTGGC-3'<br>Reverse: 5'- GAGCAGTATCTTCCATCCAC-3'	4691	This study
qRT-PCR	IDA5 (coding an actin protein; as an endogenous control)	Forward: 5'-GACATGGAGAAGATCTGGCA-3'<br>Reverse: 5'-GTCTCGAACATGATCTGGGT-3'	137	5
	PMA2	Forward: 5'- CAACACCTTCTTCGGCAGGG -3'<br>Reverse: 5'- AGCTCAATCACCACCCAGAC -3'	129	This study
	PMA3	Forward: 5'-GTCGAGTTCACCGGCAAGAA-3'<br>Reverse: 5'-GAATGAACGAGGTGACGACC-3'	133	This study
	PMA4ΔCter-V	Forward: 5'-CAACATGTACCCCTCCGCTT-3'<br>Reverse: 5'-AGCCGTCAGCCTTCTCAATC-3'	95	This study
PCR verification of gene insertion site	PMA4ΔCter-V4	Forward: 5'- GGAAGGGCGTGGAGATTGAC -3'<br>Reverse: 5'- TCCGAGGTTATTTAGTCGGGC -3'	199 (When no gene is inserted)	This study
	PMA4ΔCter-V10 D1	Forward: 5'- TCAATGCACCTCCCTTCCAC -3'<br>Reverse: 5'- TGATGCCTCCCACACCATAC -3'	1198 (When no deletion occurred)	This study
	PMA4ΔCter-V10 D2	Forward: 5'- CACGACGGACCACCATGTTA -3'<br>Reverse: 5'- ATGGCTCTTTCTGTCCCAGC -3'	1136 (When no deletion occurred)	This study

Supplementary Table 2. Annotation of genes lost as a consequence of PMAΔCter-V coding gene insertion (based on JGI *Chlamydomonas reinhardtii* v5.6).

Strain	Alias	Gene identifier	Strand	Auto define and description
PMAΔCter-V4	-	Cre07.g331114	+	-
PMAΔCter-V10	CTPA2; TSP2	Cre06.g265850	–	C-terminal peptidase, Tail-specific peptidase, possibly the D1 processing peptidase; Contains a PDZ domain
	-	Cre06.g265900	+	Putative hydrolases of HD superfamily; Metal-dependent phosphohydrolase
	DHC3	Cre06.g265950	–	Dynein heavy chain 6, axonemal; Putative flagellar inner arm dynein heavy chain. Has not been identified in Chlamydomonas flagella in mass spec proteomic study by Yagi *et al.* [PMID: 19351714]; novel minor type found exclusively at the flagellar basal portion

Supplementary Table 3. Flow cytometry statistics.

Strain	Count	Median fluorescence intensity	Mean fluorescence intensity	Fluorescence intensity standard deviation
WT	3286	6227	6529	2115
PMAΔCter-V4	3173	19514	19997	6338
PMAΔCter-V10	3100	13849	14753	6897
V8	3166	51957	52573	16408

Supplementary Table 4. Outdoor cultivation results (n = 2 biologically independent samples).

	WT	PMAΔCter-V4
Culture period (d)	14	14
OD_{800} difference	0.678±0.023	1.625±0.040
Initial pH (immediately before the seeding)	7.105±0.007	7.110±0.010
Final pH	6.645±0.007	6.480±0.010
Biomass production (mg L^{-1})	572.4±19.72	1306±31.83
Biomass productivity (mg biomass L^{-1} d^{-1})	40.88±1.408	93.30±2.273
Carbon content (wt. %)	48.60	47.43
CO_2 fixation rate (mg CO_2 L^{-1} d^{-1})	72.82±2.508	162.2±3.951
Total lipid (wt. %)	20.90±0.693	20.18±3.300
Lipid productivity (mg lipid L^{-1} d^{-1})	8.551±0.578	18.86±3.537
FAME content	6.867±0.111	14.08±0.163
Biodiesel productivity (mg FAME L^{-1} d^{-1})	2.809±0.142	13.14±0.168
LHV (lower heating value; kJ g^{-1})	18.01	17.40
Calorific productivity (kJ L^{-1} d^{-1})	0.736±0.025	1.623±0.040

Source data are provided as a Source Data file.

Supplementary references

1. Mortensen, L. M., Gislerød, H. R. The growth of *Chlamydomonas reinhardtii* as influenced by high CO_2 and low O_2 in flue gas from a silicomanganese smelter. *J. Appl. Phycol.* **27**, 633–638 (2015).
2. Harris, E. H. *The Chlamydomonas sourcebook (Second Edition) Ch. 8* (Elsevier B. V., Amsterdam, 2009)
3. Li, J., Li, C., Lan, C. Q., Liao, D. Effects of sodium bicarbonate on cell growth, lipid, accumulation, and morphology of *Chlorella vulgaris*. *Microb. Cell Fact.* **17**, 111, (2018)
4. Sung, Y. J., Choi, H. I., Lee, J. S., Hong, M. E., Sim, S. J. Screening of oleaginous algal strains from *Chlamydomonas reinhardtii* mutant libraries via density gradient centrifugation. *Biotechnol. Bioeng.* **116**, 3179–3188 (2019).
5. Tibiletti, T., Auroy, P., Peltier, G., Caffarri, S. *Chlamydomonas reinhardtii* PsbS protein is functional and accumulates rapidly and transiently under high light. *Plant Physiol.* **171**, 2717–2730 (2016).
6. VanPutte, R. D., Patterson, C. O. Microalgal plasma membranes purified by aqueous two-phase partitioning. *Trans. Ill. State Acad. Sci.* **96**, 71–86 (2003).
7. Kim, J. Y. H. *et al.* Microfluidic high-throughput selection of microalgal strains with superior photosynthetic productivity using competitive phototaxis. *Sci. Rep.* **6**, 21155 (2016)
8. Granados, M. E., Soriano, E., Saavedra-Molina, A. Use of pluronic acid F-127 with Fluo-3/AM probe to determine intracellular calcium changes elicited in bean protoplasts. *Phytochem. Anal.* **8**, 204–208 (1997).
9. Blifernez-Klassen, O. *et al.* Cellulose degradation and assimilation by the unicellular phototrophic eukaryote *Chlamydomonas reinhardtii*. *Nat. Commun.* **3**, 1214 (2012)
10. Zhang, H., Zeng, R., Chen, D., Liu, J. A pivotal role of vacuolar H^+-ATPase in regulation of lipid production in *Phaeodactylum tricornutum*. *Sci. Rep.* **6**, 31318 (2016).
11. Grant, R. L., Acosta, D. Interactions of intracellular pH and intracellular calcium in primary cultures of rabbit corneal epithelial cells. *In Vitro Cell. Dev. Biol.-Anim.* **32**, 38–45 (1996).
12. Civitelli, R., Reid, I. R., Halstead, L. R., Avioli, L. V., Hruska, K. A. Membrane potential and cation content of osteoblast-like cells (UMR 106) assessed by fluorescent dyes. *J. Cell. Physiol.*, **131**, 434–441 (1987).
13. James-Kracke, M. R. Quick and accurate method to convert BCECF fluorescence to pH_i: Calibration in three different types of cell preparations. *J. Cell. Physiol.*, **151**, 596–603 (1992).
14. Serrano, R. H^+-ATPase from plasma membranes of *Saccharomyces cerevisiae* and *Avena sativa* roots: purification and reconstitution. *Methods Enzymol.*, **157**, 533–544 (1988)
15. Oh, Y. J. *et al*. Cytochrome b5 reductase 1 triggers serial reactions that lead to iron uptake in plants. *Mol. Plant* **9**, 501–513 (2016).
16. Okumura, M. *et al.* Characterization of the plasma membrane H^+-ATPase in the liverwort *Marchantia polymorpha. Plant Physiol.* **159**, 826–834 (2012).
