# Supplementary material for: Augmented CO2 tolerance by expressing a single H+-pump enables microalgal valorization of industrial flue gas
Source: Nat Commun. 2021 Oct 18;12:6049. doi: 10.1038/s41467-021-26325-5 (PMC8523702; doi:10.1038/s41467-021-26325-5)
Supplement: Supplementary file 3 — Description of Additional Supplementary Files [file 41467_2021_26325_MOESM3_ESM.pdf]

Description of Additional Supplementary Files

File Name: Supplementary Data 1

Description: This includes all the data associated with the RNA-Seq which were derived by the generalized linear model (GLM) likelihood ratio (LR) test.

File Name: Supplementary Data 2

Description: This includes the results associated with the Gene Ontology (GO) enrichment analysis of biological processes. Fisher's exact test method was used to determine the statistical significance ($p < 0.05$).

File Name: Supplementary Data 3

Description: This includes the information associated with the genes of interest for elucidating the cellular response to extremely high CO_2 conditions. The descriptions and roles of the genes are based on the indicated references (provided in the Supplementary Dataset 3 file) and JGI Genome Portal (*Chlamydomonas reinhardtii* v5.6).
