# Supplementary material for: Augmented CO2 tolerance by expressing a single H+-pump enables microalgal valorization of industrial flue gas
Source: Nat Commun. 2021 Oct 18;12:6049. doi: 10.1038/s41467-021-26325-5 (PMC8523702; doi:10.1038/s41467-021-26325-5)
Supplement: Supplementary file 7 — Reporting Summary [file 41467_2021_26325_MOESM7_ESM.pdf]

nature portfolio

Corresponding author(s): Sang Jun Sim

Last updated by author(s): Sep 27, 2021

Reporting Summary

Nature Portfolio wishes to improve the reproducibility of the work that we publish. This form provides structure for consistency and transparency in reporting. For further information on Nature Portfolio policies, see our Editorial Policies and the Editorial Policy Checklist.

Statistics

For all statistical analyses, confirm that the following items are present in the figure legend, table legend, main text, or Methods section.

n/a	Confirmed	
☐	☒	The exact sample size (*n*) for each experimental group/condition, given as a discrete number and unit of measurement
☐	☒	A statement on whether measurements were taken from distinct samples or whether the same sample was measured repeatedly
☐	☒	The statistical test(s) used AND whether they are one- or two-sided<br>*Only common tests should be described solely by name; describe more complex techniques in the Methods section.*
☐	☒	A description of all covariates tested
☒	☐	A description of any assumptions or corrections, such as tests of normality and adjustment for multiple comparisons
☐	☒	A full description of the statistical parameters including central tendency (e.g. means) or other basic estimates (e.g. regression coefficient) AND variation (e.g. standard deviation) or associated estimates of uncertainty (e.g. confidence intervals)
☐	☒	For null hypothesis testing, the test statistic (e.g. *F*, *t*, *r*) with confidence intervals, effect sizes, degrees of freedom and *P* value noted<br>*Give P values as exact values whenever suitable.*
☒	☐	For Bayesian analysis, information on the choice of priors and Markov chain Monte Carlo settings
☒	☐	For hierarchical and complex designs, identification of the appropriate level for tests and full reporting of outcomes
☒	☐	Estimates of effect sizes (e.g. Cohen's *d*, Pearson's *r*), indicating how they were calculated

Our web collection on statistics for biologists contains articles on many of the points above.

Software and code

Policy information about availability of computer code

Data collection	The updated Chlamydomonas reinhardtii genome and gene annotation for the RNA sequencing and DNA insertion analysis were downloaded from JGI Phytozome v13, Chlamydomonas reinhardtii v5.6 [https://data.jgi.doe.gov/refine-download/phytozome?organism=Creinhardtii&expanded=281]. The RNA sequencing was performed using the Illumina NovaSeq 6000 sequencing platform with PE150 mode. For data collection in the flow cytometry, BD Accuri C6 Plus software (v1.0.23.1) was used.
Data analysis	For the RNA sequencing and transgene insertion site analysis in this study, open source programs, Bowtie2 (v2.2.6) and HISAT2 (v2.2.1), were used. R/Bioconductor (v3.12) packages, including topGO (v3.28.1) and edgeR (v2.38.1), were used to analyze the relative expression. For image analysis, ImageJ software (v1.53k) was used. For analyzing flow cytometry data, Flowjo software (v10.7.1) was used.

For manuscripts utilizing custom algorithms or software that are central to the research but not yet described in published literature, software must be made available to editors and reviewers. We strongly encourage code deposition in a community repository (e.g. GitHub). See the Nature Portfolio guidelines for submitting code & software for further information.

Data

Policy information about availability of data

All manuscripts must include a data availability statement. This statement should provide the following information, where applicable:

- Accession codes, unique identifiers, or web links for publicly available datasets
- A description of any restrictions on data availability
- For clinical datasets or third party data, please ensure that the statement adheres to our policy

Data supporting the findings of this work are available within the paper and its Supplementary Information files. Source data, including raw data and original images, are provided with this paper. A reporting summary for this article is available as a Supplementary Information file. All RNA sequencing data from this study have

been deposited into the NCBI Sequence Read Archive (SRA) [http://www.ncbi.nlm.nih.gov/sra/] under BioProject accession code PRJNA720171 [https://www.ncbi.nlm.nih.gov/bioproject/PRJNA720171]. Details of the relative expression are also provided in Figure 1 and Supplementary Dataset 1. The updated Chlamydomonas reinhardtii genome and gene annotation for the RNA sequencing and DNA insertion analysis were downloaded from JGI Phytozome v13, Chlamydomonas reinhardtii v5.6 [https://data.jgi.doe.gov/refine-download/phytozome?organism=Creinhardtii&expanded=281]. All sequence reads for the insertion site were deposited in the NCBI SRA with BioProject accession code PRJNA767110 [https://www.ncbi.nlm.nih.gov/bioproject/PRJNA767110]. All the relevant data are available from the corresponding author upon reasonable request. The related statement is included in the manuscript.

Field-specific reporting

Please select the one below that is the best fit for your research. If you are not sure, read the appropriate sections before making your selection.

☒ Life sciences ☐ Behavioural & social sciences ☐ Ecological, evolutionary & environmental sciences

For a reference copy of the document with all sections, see nature.com/documents/nr-reporting-summary-flat.pdf

Life sciences study design

All studies must disclose on these points even when the disclosure is negative.

Sample size	No sample size was predetermined. For the RNA sequencing, a pool of total RNA from six biological replicates (cells in each culture were totally harvested) was sequenced for each three condition (i.e., ambient (atmospheric), pH 5.0 acidic, and 20% high CO2 conditions), respectively, to reduce the amount of biological variability. For experiments involving RT-PCR, growth assessment, photosynthetic rate assay, ELISA, intracellular pH measurement, in vitro ATPase activity assay, etc., n=2 was chosen as the minimal replicate number. For the flow cytometry analysis, the fluorescence measurement lasted until 10,000 events occurred. Sample size was determined to be adequate based on the magnitude and consistency of measurable differences among groups.
Data exclusions	No data were excluded from the analyses.
Replication	All experiments were performed with at least two biological replicates, including RT-PCR, growth assessment, photosynthetic rate assay, ELISA, etc. Two quantitative biochemical experiments, including intracellular pH measurement and in vitro ATPase activity assay (ATPase-mediated ATP hydrolysis performance test), were conducted in quadruplicate to secure the reliability (indicated in the manuscript and the corresponding legends). All replication attempts were successful.
Randomization	No specific method of randomization was used for the generation of samples or cell experiments since all algal cells in each genotype are identical. For any experiments in which aliquots are needed, they were sampled randomly from each cell line culture. Confocal imaging were selected randomly from three biologically independent samples.
Blinding	Blinding was not used in this study. The investigators were not blinded during data collection and analyses.

Reporting for specific materials, systems and methods

We require information from authors about some types of materials, experimental systems and methods used in many studies. Here, indicate whether each material, system or method listed is relevant to your study. If you are not sure if a list item applies to your research, read the appropriate section before selecting a response.

Materials & experimental systems

n/a	Involved in the study	
☐	☒	Antibodies
☐	☒	Eukaryotic cell lines
☒	☐	Palaeontology and archaeology
☒	☐	Animals and other organisms
☒	☐	Human research participants
☒	☐	Clinical data
☒	☐	Dual use research of concern

Methods

n/a	Involved in the study	
☒	☐	ChIP-seq
☐	☒	Flow cytometry
☒	☐	MRI-based neuroimaging

Antibodies

Antibodies used	In this study, anti-plasma membrane H+-ATPase (Supplier: Agrisera, Sweden; Catalog number: AS07 260; Clonality: Polyclonal; Host: Rabbit) was used as the primary antibody for detecting the heterologously expressed PMA protein and natively expressed PMA with Western blotting at the same time. Anti-beta subunit of ATP synthase (Supplier: Agrisera, Sweden; Catalog number: AS05 085; Clonality: Polyclonal; Host: Rabbit) was used as the primary antibody for detecting the endogenous loading control (i.e., beta subunit of ATP synthase). For both cases, HRP conjugated goat anti-rabbit IgG (H+L) secondary antibody (Supplier: Agrisera, Sweden; Catalog number: AS09 602) was used as the secondary antibody.
Validation	Cross reactivity of anti-plasma membrane H+-ATPase (PMA) between those from Nicotiana tabacum and Nicotiana plumbaginifolia was confirmed by a previous immunoblotting study (Chen et al., J. Exp. Bot., 61, 1853-1867, 2010). Reactivity of the antibody towards

Nicotiana tabacum's PMA and C. reinhardtii's PMA are specified in the manufacturer's website (https://www.agrisera.com/en/artiklar/hatpase-plasma-membrane-hatpase.html) and several previous studies (Wang et al., Proc. Natl. Acad. Sci. U.S.A., 113, 12586-12591, 2016 and Yamano et al., Proc. Natl. Acad. Sci. U.S.A., 112, 7315-7320, 2015). Meanwhile, reactivity of anti-beta subunit of ATP synthase for the corresponding protein in Chlamydomonas reinhardtii was validated from a previous study (Picariello et al., PLoS ONE, 15, e0232594, 2020).

Eukaryotic cell lines

Policy information about cell lines

Cell line source(s)	The parental cell line in this study, Chlamydomonas reinhardtii CC125, was obtained from the Chlamydomonas Resource Center at the University of Minnesota.
Authentication	The cell lines were not further authenticated.
Mycoplasma contamination	The cell lines were not tested for mycoplasma contamination.
Commonly misidentified lines (See ICLAC register)	The CC125 cell line used in this study is not listed in the database.

Flow Cytometry

Plots

Confirm that:

- [x] The axis labels state the marker and fluorochrome used (e.g. CD4-FITC).
- [x] The axis scales are clearly visible. Include numbers along axes only for bottom left plot of group (a 'group' is an analysis of identical markers).
- [x] All plots are contour plots with outliers or pseudocolor plots.
- [x] A numerical value for number of cells or percentage (with statistics) is provided.

Methodology

Sample preparation	Every cell line was freshly grown prior to the flow cytometry. The cells were harvested by centrifugation until they reached their exponential growth phase (cell density of 1.5 million cells per mL). The harvested cell pellet was resuspended in freshly prepared medium. Fluorescence detection was performed without any staining process because the experiment targeted the detection of intracellularly expressed fluorescent protein.
Instrument	BD Accuri C6 Plus flow cytometer was used to measure the fluorescence.
Software	To control the flow cytometer, BD Accuri C6 Plus Software was used. For statistically analyzing the gathered data, FlowJo software was used.
Cell population abundance	The flow cytometry analysis lasted until 10,000 single-cell events occurred with the prepared cell sample. Among the events, ca. 3,000 events which was gated with the following gating strategy were analyzed. Details regarding cell population for the analysis are provided in Supplementary Table 3.
Gating strategy	The abovementioned 3,000 events were tightly gated based on the forward (FSC-A) versus side scatter (SSC-A) profiles that covered the most densely populated region of each cell line and the fluorescence populations were analyzed by plotting histogram. The fluorescence of the cells were excited with a 488 nm laser while the emission was detected using a 533/30 nm bandpass filter (FITC-A).

- [x] Tick this box to confirm that a figure exemplifying the gating strategy is provided in the Supplementary Information.
